# Apatite *c*-axis Orientation Tracks Disc-Mediated Local Principal Stress Directions in the Human Lumbar Vertebra

**DOI:** 10.1007/s00223-026-01629-0

**Published:** 2026-10-23

**Authors:** Toko Mori, Tadaaki Matsuzaka, Aira Matsugaki, Sayaka Miyabe, Nobuhito Nango, Yukihide Minoda, Hiroyoshi Iwaki, Akio Kobayashi, Takayoshi Nakano

**Affiliations:** 1 Division of Materials and Manufacturing Science, Graduate School of Engineering, The University of Osaka, 2-1, Yamadaoka, Suita, Osaka 565-0871 Japan; 2 R&D Department, Ratoc System Engineering Co., Ltd, Tokyo, Japan; 3 Department of Orthopaedic Surgery, Graduate School of Medicine, Osaka Metropolitan University, Osaka, Japan; 4 Department of Orthopaedic Surgery, Osaka Orthopedic Hospital, Osaka, Japan; 5 Department of Orthopaedic Surgery, Shiraniwa Hospital, Nara, Japan

**Keywords:** Human spine vertebrae, BAp orientation, Mechanoadaptation, Finite element analysis (FEA), Principal stress

## Abstract

Bone mechanical competence depends not only on bone mass but also on the anisotropic orientation of the collagen/biological apatite (BAp) composite, yet its relationship to the local mechanical environment in human vertebrae remains unclear. Here, we examined whether region-specific BAp *c*-axis orientation corresponds to predominant principal stress direction estimated by µCT-based finite element analysis. The anterior region showed predominantly uniaxial alignment along the craniocaudal axis, corresponding to a predominant principal stress direction oriented primarily along the craniocaudal axis. In contrast, the cranial and caudal subchondral regions exhibited a two-dimensional in-plane orientation pattern corresponding to predominant principal stress directions within the anteroposterior–mediolateral plane, consistent with load transfer through the intervertebral disc, which imposes transverse in-plane loading on the endplate region. At the corticotrabecular junction, the preferred BAp orientation changed markedly within a spatial range of 100 μm or less. Overall, these findings demonstrate region-specific stress–orientation correspondence in human vertebrae and support crystallographic orientation as a quantitative bone-quality indicator of vertebral mechanical adaptation.

**Supplementary Information:**

The online version contains supplementary material available at https://doi.org/10.1007/s00223-026-01629-0.

## Introduction

The vertebral column serves a crucial function in supporting and distributing weight [1]. Vertebrae possess a hierarchical microstructure that enables efficient load bearing; at the macroscale, they consist of dense cortical bone and trabecular bone with a three-dimensional lattice architecture, and these structures are connected by the soft intervertebral discs [2, 3]. At micro- to nanoscale levels, bone mechanical competence depends not only on bone mass but also on the anisotropic alignment of the collagen/apatite composite, in which the apatite is a key determinant of the directional mechanical properties of bone [4]. Consistently, apatite crystal orientation has been shown to substantially contribute to the elastic anisotropy of human femoral diaphyseal cortical bone [5]. BAp orientation has been quantified mainly by X-ray diffraction or by ultrasound-based measurements of the speed of sound (*SoS*) [6, 7], and previous studies have shown that it varies with bone type, morphology, and anatomical location, often in accordance with local mechanical demand [8]. These observations suggest that crystallographic orientation may serve as a sensitive indicator of the local mechanical environment. Such orientation–mechanics relationships may also be altered under pathological conditions; for example, altered BAp orientation has been reported in osteoporotic animal models [9, 10]. In contrast to the diaphysis of long bones, vertebrae are subjected to region-dependent local mechanical environments generated by complex load transmission through the intervertebral discs and endplates. However, quantitative three-dimensional characterization of BAp orientation in human vertebral cortical and subchondral regions remains limited, and the relationship between local stress direction and crystallographic orientation has not been fully clarified.

Here, we examined whether region-specific BAp *c*-axis orientation corresponds to local principal stress distribution in human vertebrae. We quantitatively analyzed the three-directional distribution of BAp *c*-axis orientation in the cortical and subchondral regions using microbeam X-ray diffraction and compared these data with predominant principal stress directions derived from µCT-based finite element models reconstructed from the corresponding vertebral specimens. This study provides evidence of region-specific stress–orientation correspondence in human vertebrae and may inform bone-quality–oriented structural reconstruction, device design, and future in vivo assessment of anisotropic bone quality, including molecular regulators of crystallographic alignment.

## Materials and Methods

### Human Vertebral Body Samples

**Table 1:** Human lumbar spine (L4) specimens

Specimen No.	Sex	Age (years)	DEXA-BMD (g/cm^2^)
1	Male	66	0.78
2	Male	68	0.76
3	Female	70	0.40
4	Female	86	0.35

Sex indicates biological sex (male/female). DEXA-BMD is bone mineral density by dual energy X-ray absorptiometry method

Four lumbar vertebrae specimens were obtained from male and female donors aged 66–86 years. This study was reviewed and approved by the Research Ethics Committee of Osaka Prefecture University Hospital [11]. BMD for each vertebra was measured using a dual-energy X-ray absorptiometry bone densitometer (DCS-600EX, ALOKA Co.) (Table 1). The extracted L4 vertebrae were sectioned along the midsagittal plane, fixed in 10% neutral-buffered formalin, and polished to prepare a single midsagittal slice with a final thickness of 1.25 mm from each vertebral body. In this study, BAp *c*-axis and trabecular alignment in the sagittal plane were defined with the anteroposterior direction as 0°, and the craniocaudal axis as 90°.

### Quantitative Analysis of BAp Orientation in Vertebrae Using Microbeam X-Ray Diffractometer

#### Region-Specific Three-Dimensional Assessment of BAp Orientation in Cortical Bone

The *c*-axis orientation of BAp crystals was analyzed using transmission- and reflection-type microbeam X-ray diffraction systems. The sagittal plane of each vertebral body was divided into a 2.5 mm grid, and XRD measurement points, indicated by the red dots in Fig. S1, were defined along the cortical contour in the anterior, caudal, and cranial regions. Because the XRD measurement points were defined at 2.5 mm intervals along the cortical contour, the number of measurement points varied according to the size and morphology of each vertebral body. Across specimens, 7–10 points were measured in the anterior region, 8–13 in the cranial region, and 7–10 in the caudal region, corresponding to a total of 24–33 XRD measurement points per specimen.

For transmission measurements, a transmission-type X-ray diffraction system (R-AXIS BQ, Rigaku Co.) was used with MoKα radiation at 50 kV and 90 mA. The incident beam was focused to a 300 μm-diameter spot using a metal collimator and irradiated perpendicularly to each measurement point. Diffraction profiles were recorded at 1° intervals to obtain the angular distribution of BAp orientation within the sagittal plane. At each angle, preferential *c*-axis orientation was quantified by the integrated intensity ratio (002)/(310). Non-oriented hydroxyapatite powder (NIST #2910) exhibited an integrated intensity ratio of 0.6, so values above this level were regarded as indicating preferential orientation.

To assess orientation along the mediolateral direction, which is normal to the sagittal plane, a reflection-type microbeam XRD system (D8 Discover with GADDS, Bruker AXS) was employed at the same measurement points. CuKα radiation (λ = 1.5418 Å) was operated at 45 kV and 110 mA. The BAp orientation value along the mediolateral direction was quantified by the integrated intensity ratio (002)/(310).

For the transmission-type measurements, the angular distribution of the intensity ratio was fitted to an elliptical curve using Eq. (1):

1 $$\:\begin{array}{c}r=\frac{ab}{\sqrt{{a}^{2}{\mathrm{sin}}^{2}\left(\theta\:-\alpha\:\right)+{b}^{2}{\mathrm{cos}}^{2}\left(\theta\:-\alpha\:\right)}}-c \end{array}$$

The parameters *a*, *b*, *c*, and *α* were determined simultaneously by least-squares fitting to the complete angular distribution of the measured intensity ratios. Here, *a* and *b* represent the major and minor radii of the fitted elliptical distribution, respectively, *α* represents the angular direction of the major axis, and *c* is a constant. In the polar representation, the radial coordinate *r*_*(θ)*_ represents the fitted (002)/(310) intensity ratio at measurement angle *θ*; thus, the fitted distribution represents the angular dependence of the diffraction intensity ratio rather than the physical shape of the apatite crystals. The maximum value of the fitted angular distribution (*r*_*max*_) was defined as the BAp orientation value at each measurement point, and the corresponding angle was defined as the preferred BAp *c*-axis direction. When *r* did not vary with *θ*, no unique preferred direction could be defined within the measured plane. A schematic explanation of the polar fitting and representative distributions with different BAp orientation values are provided in Fig. S2.

To compare the preferred BAp *c*-axis orientation with the local cortical surface orientation, the local cortical surface direction at each XRD measurement position was determined manually using ImageJ (National Institutes of Health, Bethesda, MD, USA) from the CCD camera image acquired at the corresponding X-ray beam irradiation position during the XRD measurement. In the sagittal plane, the posterior direction perpendicular to the craniocaudal axis was defined as 0°, and the superior direction along the craniocaudal axis as 90° for both BAp *c*-axis orientation and local cortical surface orientation.

#### Analysis of Local BAp Orientation Across the Corticotrabecular Junction

The BAp *c*-axis orientation across the caudal corticotrabecular junction of Specimen 1 was analyzed at 100 μm intervals over a 1 mm-wide region spanning the endplate-adjacent cortical region and the adjacent trabecular bone within the subchondral region. Using the same diffraction system (R-AXIS BQ, Rigaku Co.), the incident beam was focused into a 100 μm-diameter circular spot using a metallic collimator. At each measurement position, the angular distribution of the (002)/(310) intensity ratio was analyzed using the same procedure described above to determine the preferred BAp *c*-axis orientation and the maximum BAp orientation value.

For graphical representation, the angular distribution of the (002)/(310) intensity ratio at each measurement position was fitted using the elliptical function described above. The black ellipse represents the fitted angular distribution, and the red line indicates the preferred BAp *c*-axis direction corresponding to the major axis of the fitted ellipse. The concentric circles represent the (002)/(310) intensity ratio scale. The numerical value shown for each measurement position represents the maximum fitted the intensity ratio of (002)/(310).

### Analysis of Principal Stress Distribution of Vertebral Bone Using the Finite Element Method

#### Finite Element Analysis (FEA) Based on X-Ray Imaging

CT imaging of the L4 vertebra was performed using an X-ray µCT system (SMX-100CT-SV, Shimadzu) at 70 kV and 50 µA. The CT image resolution ranged from 77 to 162 μm/pixel among specimens. The reconstructed images were subsequently used to generate three-dimensional voxel-based FE models with an isotropic voxel size of approximately 147 μm. CT datasets were processed using TRI/3D-BON (Ratoc System Engineering Co., Ltd., Tokyo, Japan). Calibration was performed using a hydroxyapatite-based bone mineral phantom (Ratoc System Engineering Co., Ltd.), and CT values were converted to tissue mineral density (TMD)-equivalent values (mg/cm³). Based on the calibrated density values, each voxel was categorized into cortical bone (≥ 690 mg/cm³), trabecular bone (395–690 mg/cm³), or low-density region (< 395 mg/cm³). The thresholds of 690 and 395 mg/cm³ were selected with reference to density-based criteria previously used in quantitative CT analyses of bone and were applied here to define discrete density-based voxel classes for FE material assignment [12]. These categories represent density-defined voxel classes rather than apparent structural averages.

Representative material properties were assigned discretely to each category (Table S1). The Young’s modulus assigned to cortical bone (22 GPa) is within the reported range for human cortical bone, and trabecular bone modulus (3.5 GPa) represents tissue-level vertebral trabecular bone [13, 14]. The modulus assigned to the low-density region (0.44 GPa) was introduced as a low-stiffness filler for voxels below the trabecular threshold and does not imply soft biological tissue [15]. Material properties were not continuously scaled to local density. Because the objective was to evaluate regional stress patterns and principal stress directions rather than exact absolute stress magnitudes, a simplified discrete material assignment was adopted. Under linear-elastic assumptions, principal stress direction is expected to be less sensitive than stress magnitude to moderate modulus variation. Finite element analysis was performed using TRI/3D-FEM (Ratoc System Engineering Co., Ltd.). The FE model consisted of a single L4 vertebral body with portions of the two adjacent intervertebral discs, the L3/L4 disc on the cranial side and the L4/L5 disc on the caudal side. The disc geometries were not reconstructed from donor-specific imaging data but were artificially created using the modeling function of TRI/3D-FEM so that they conformed without gaps to the corresponding cranial and caudal endplate surfaces of L4. Each intervertebral disc was divided along its height, and only the half adjacent to L4 was included in the FE model. At the disc–endplate interfaces, adjacent disc and vertebral elements shared nodes, thereby ensuring displacement continuity and direct load transfer across the interfaces. The disc segments were modeled as homogeneous, isotropic, linear-elastic materials [16, 17], with a Young’s modulus of 8 MPa [18] and a Poisson’s ratio of 0.45 [19], while the vertebral body was assigned a Poisson’s ratio of 0.30 [20]. A loading support was positioned cranial to the L3/L4 disc segment, and a fixing support was positioned caudal to the L4/L5 disc segment. A compressive load of 500 N was applied in the craniocaudal direction to the upper surface of the loading support, simulating physiological axial loading in a standing posture [21, 22]. The lower surface of the fixing support was fully constrained. The load was thereby transferred to the L4 vertebral body through the adjacent disc segments. Posterior elements of spine were excluded due to their minimal contribution to axial compressive load support [23].

#### Principal Stress Analysis and Directional Comparison with BAp Orientation

The whole-vertebra FE model was used to calculate the three-dimensional stress field throughout the vertebral body. Because the FE model was constructed directly from the µCT dataset of the same vertebral specimen, the coordinates of the individual XRD measurement positions were identified on the µCT images and registered to the corresponding coordinates in the FE model. At each registered position, a local FE region of 3 × 3 × 9 voxels (81 voxels; approximately 0.44 × 0.44 × 1.32 mm) was defined for site-matched analysis. This region was selected with consideration of the 300-µm X-ray beam diameter and the 1.25 mm specimen thickness and was used instead of a single voxel to reduce the influence of small positional discrepancies between the XRD irradiation position and the FE discretization.

The six stress components (S_xx_, S_yy_, S_zz_, S_xy_, S_xz_, and S_yz_) were averaged within each local FE region to construct a representative local stress tensor. The three principal stresses were determined as the eigenvalues of this tensor, and the maximum (*σ*_1_) and minimum (*σ*_3_) principal stresses were defined as the largest and smallest eigenvalues, respectively. Their corresponding principal directions were defined by the associated eigenvectors. In the present analysis, tensile and compressive states were defined based on the sign of the principal stress (positive for tensile, negative for compressive). Of the maximum and minimum principal stresses, the one with the greater absolute magnitude was defined as the predominant principal stress, and its associated eigenvector was defined as the predominant principal stress direction.

For the three-directional analysis in the cranial and caudal regions, the predominant principal stress was resolved into the anteroposterior, craniocaudal, and mediolateral components. These components were averaged across the XRD-corresponding FE locations within each region for each vertebra, and the mean and SD were subsequently calculated across the four vertebrae (*N* = 4).

For the point-by-point comparison with BAp orientation measured by transmission-type XRD, the three-dimensional predominant principal stress direction was orthogonally projected onto the sagittal plane. The predominant principal stress direction was represented as a unit vector before projection; therefore, the length of the projected vector represents the magnitude of its sagittal-plane component rather than the principal stress magnitude. The projected direction was compared with the preferred BAp direction at the corresponding XRD measurement location, and their angular deviation (Δ*θ*) was calculated.

### Statistical Analysis

For inferential statistical comparisons, each vertebra was treated as the independent statistical unit (*N* = 4), and individual measurement points were not pooled across specimens as independent observations. Measurements were summarized within each specimen according to the anatomical region and analysis type before statistical testing. For the three-directional analysis of BAp orientation, two-way repeated-measures ANOVA was performed with anatomical region (anterior, cranial, and caudal) and direction (mediolateral, anteroposterior, and craniocaudal) as within-specimen factors. Following a significant interaction between anatomical region and direction, pairwise comparisons were performed among directions within each anatomical region and among anatomical regions within each direction, with adjustment for multiple comparisons. For the three-directional analysis of predominant principal stress, two-way repeated-measures ANOVA was performed with anatomical region (cranial and caudal) and direction (mediolateral, anteroposterior, and craniocaudal) as within-specimen factors. Following a significant interaction between anatomical region and direction, planned pairwise comparisons of the anteroposterior and mediolateral components with the craniocaudal component were performed separately within each anatomical region, with Holm adjustment for the two comparisons. No pairwise comparisons were performed between the cranial and caudal regions. BAp orientation values along the anteroposterior and craniocaudal directions were evaluated by transmission-type XRD within the sagittal plane, whereas the mediolateral value was obtained independently by reflection-type XRD along the direction normal to the sagittal plane. The directional components of the predominant principal stress along the same three anatomical directions were obtained by resolving the three-dimensional predominant principal stress into the anteroposterior, craniocaudal, and mediolateral components. The probability value of *P* < 0.05 was considered statistically significant. Because orientation data represent axial data (0° ≡ 180°), mean orientation values were calculated using doubled-angle circular statistics within the range of − 90° to 90°. Angular deviation (Δ*θ*) was defined as the smaller angular difference between two axial directions and therefore ranged from 0° to 90°, with smaller values indicating closer directional correspondence. For plots comparing preferred BAp orientation with either cortical direction or predominant principal stress direction, values at each measurement position were first averaged across specimens to avoid treating repeated measurements as independent observations. In contrast, for analyses of angular deviation (Δ*θ*), Δ*θ* values were first calculated at each measurement position and then averaged within each specimen, such that the specimen was treated as the unit of analysis.

## Results

### BAp Crystal Orientation in the Cortical Bone of Vertebral Bodies

#### Vertebral Cortical Bone Exhibited Distinct Region-Specific Patterns of BAp *c*-Axis Orientation

**Fig. 1:**
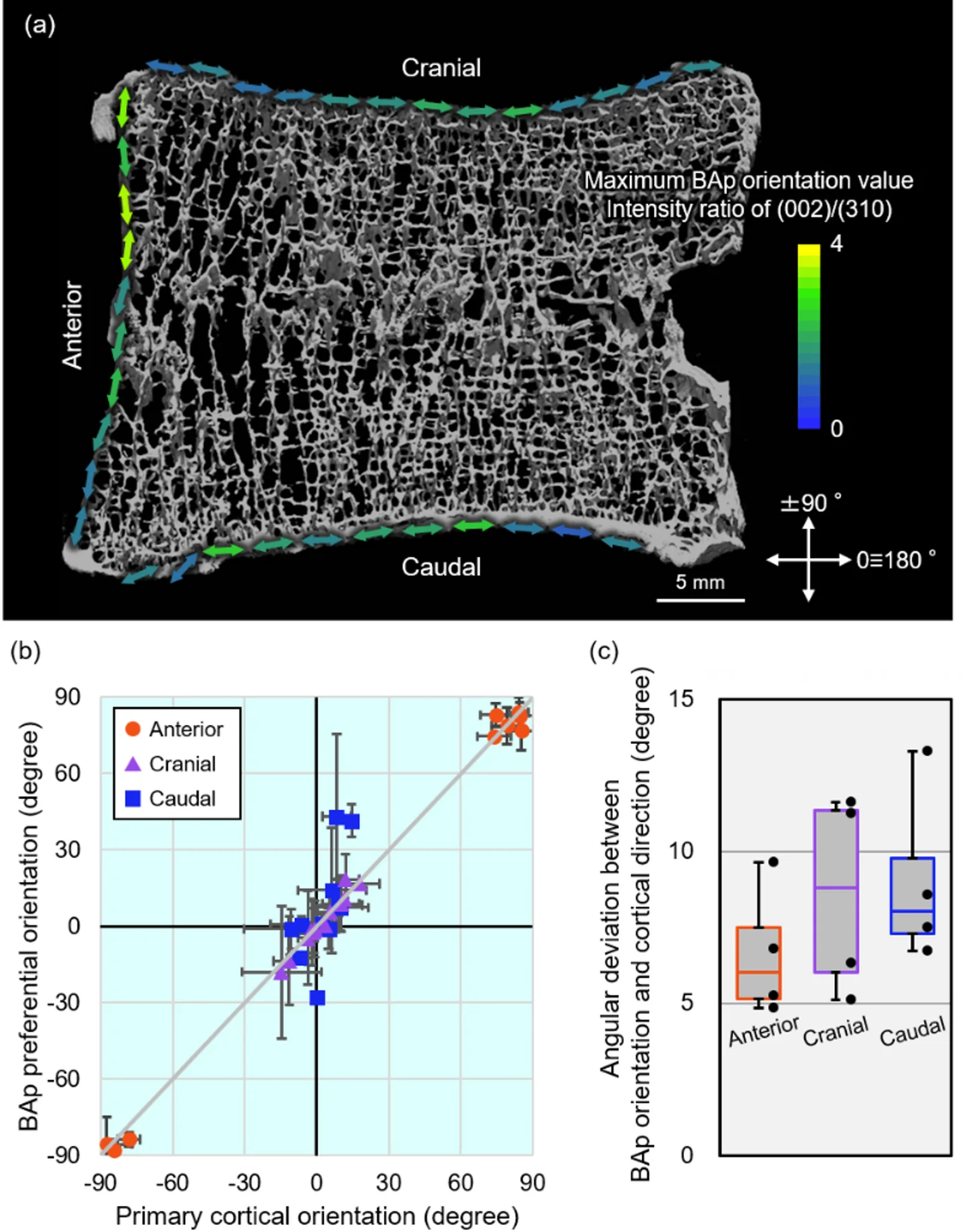
Regional correspondence between preferred BAp *c*-axis orientation and local cortical surface orientation. **a** Preferred BAp *c*-axis orientation and maximum orientation value in a representative specimen (Specimen 1). Bidirectional arrows indicate preferred orientation, and arrow color indicates the maximum intensity ratio of (002)/(310) value. Because orientation is axial, 0° ≡ 180° and + 90° ≡ −90°. **b** Relationship between preferred BAp orientation and local cortical surface orientation. Each point represents the mean across four specimens at the corresponding measurement position; error bars indicate SD. The gray line indicates y = x. **c** Angular deviation (Δ*θ*) between preferred BAp orientation and local cortical surface orientation (*N* = 4). Smaller values indicate closer directional correspondence

The preferred BAp *c*-axis orientations and corresponding maximum BAp orientation values in the representative specimen (Specimen 1) are shown in Fig. 1a. The anterior region showed higher maximum BAp orientation values than the cranial and caudal regions. The preferred crystal direction at each measurement point generally aligned parallel to the local cortical surface in the sagittal plane: craniocaudal alignment predominated along the anterior surface, whereas anteroposterior alignment predominated along the cranial and caudal surfaces. This region-specific pattern was consistent across the four specimens.

To quantitatively assess this correspondence, preferred BAp orientation was compared with local cortical surface orientation at corresponding measurement positions. In Fig. 1b, the specimen-averaged values at each measurement position were distributed close to the identity line, indicating descriptive positional correspondence between preferred BAp orientation and local cortical surface orientation. Consistently, the angular deviation (Δ*θ*) between BAp orientation and local cortical surface orientation remained relatively small in each region (Fig. 1c).

**Fig. 2:**
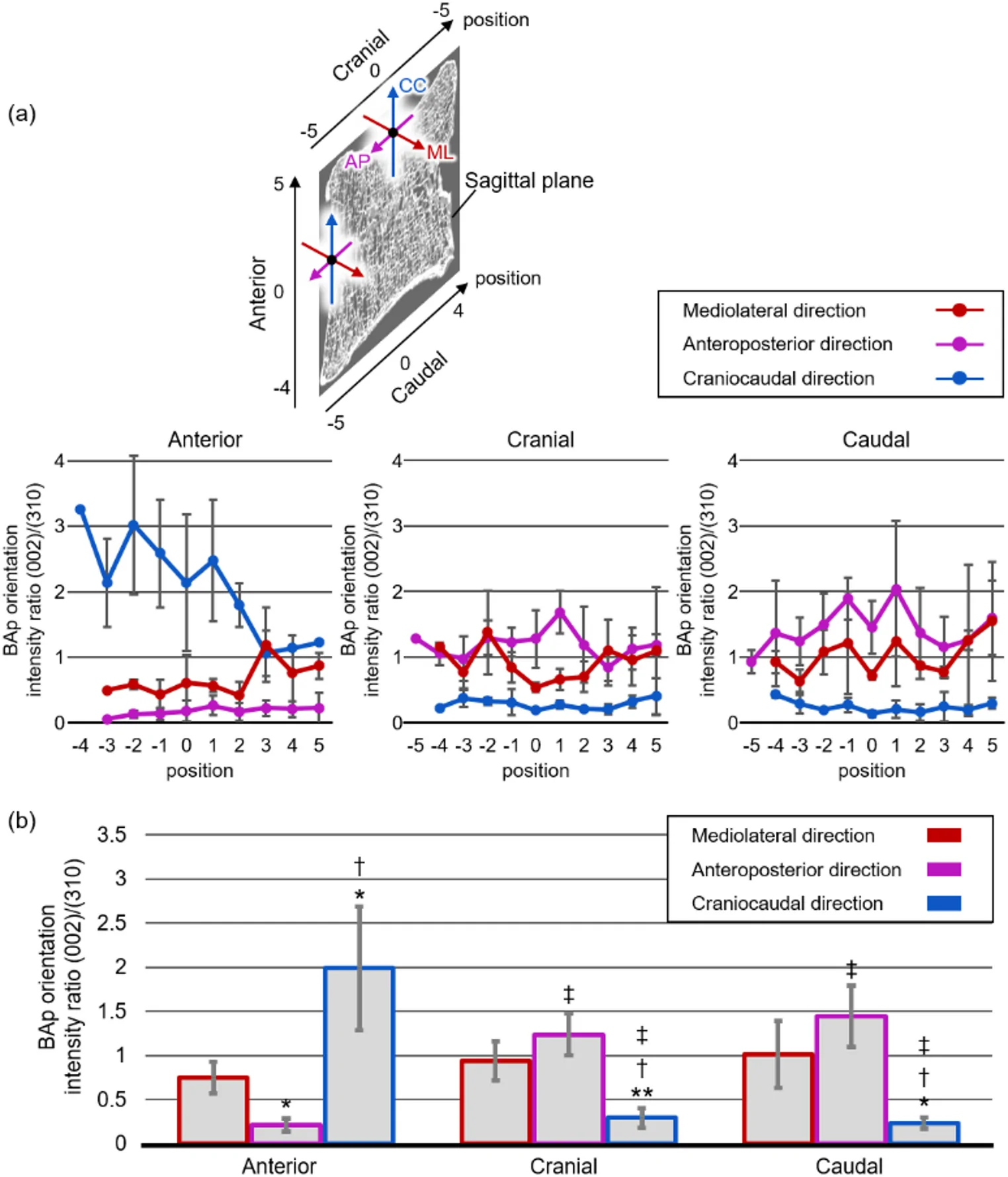
**a** BAp orientation values along the anteroposterior, craniocaudal, and mediolateral axes in the anterior, cranial, and caudal regions (*N* = 4). Data are presented as mean ± SD across the four specimens at each corresponding measurement position. **b** Comparison of BAp orientation values across regions and axes (*N* = 4). The anterior region showed predominant craniocaudal alignment, whereas the cranial and caudal regions showed two-dimensional alignment within the anteroposterior–mediolateral plane. Statistical differences in (**b**) were evaluated by two-way repeated-measures ANOVA with anatomical region and direction as within-specimen factors, followed by pairwise comparisons with adjustment for multiple comparisons. No significant differences were detected between the cranial and caudal regions in any of the three directions, or among the three regions in the mediolateral direction. *: *P* < 0.05, **: *P* < 0.01 vs. mediolateral direction within the same region. †: *P* < 0.05 vs. anteroposterior direction within the same region. ‡: *P* < 0.05 vs. the corresponding direction in the anterior region

Reflection-type microbeam XRD was then used to assess BAp orientation along the mediolateral direction, providing three-directional orientation information together with the transmission-type measurements. Based on the region-specific orientation pattern, the anterior region was interpreted as showing maximum orientation along the craniocaudal axis and minimum orientation along the anteroposterior axis within the sagittal plane, whereas the cranial and caudal regions showed the opposite relationship. Because reflection-type XRD probes the direction perpendicular to the sagittal plane, it represents the mediolateral axis. Figure 2 summarizes BAp orientation values along the mediolateral, anteroposterior, and craniocaudal axes. Because XRD measurements were performed at 2.5 mm intervals along the cortical contour, the number of measurement positions differed among the anterior, cranial, and caudal regions owing to differences in vertebral morphology and regional dimensions. Two-way repeated measures ANOVA revealed a significant interaction between anatomical region and direction (*P* < 0.001). In the anterior region, craniocaudal orientation was significantly higher than anteroposterior and mediolateral orientation, and mediolateral orientation was significantly higher than anteroposterior orientation. In contrast, in the cranial and caudal regions, anteroposterior and mediolateral orientations were comparable and significantly higher than craniocaudal orientation. For between-region comparisons, anteroposterior and craniocaudal orientation values differed significantly between the anterior region and the cranial and caudal regions, whereas no significant differences were detected between the cranial and caudal regions for any of the three directions. No significant regional differences were observed in the mediolateral orientation values. These results indicate predominantly uniaxial BAp orientation along the craniocaudal axis in the anterior region and a two-dimensional orientation pattern within the anteroposterior–mediolateral plane in the cranial and caudal regions.

### Microscale Changes in BAp Orientation Were Observed at the Corticotrabecular Junction Beneath the Intervertebral Disc

**Fig. 3:**
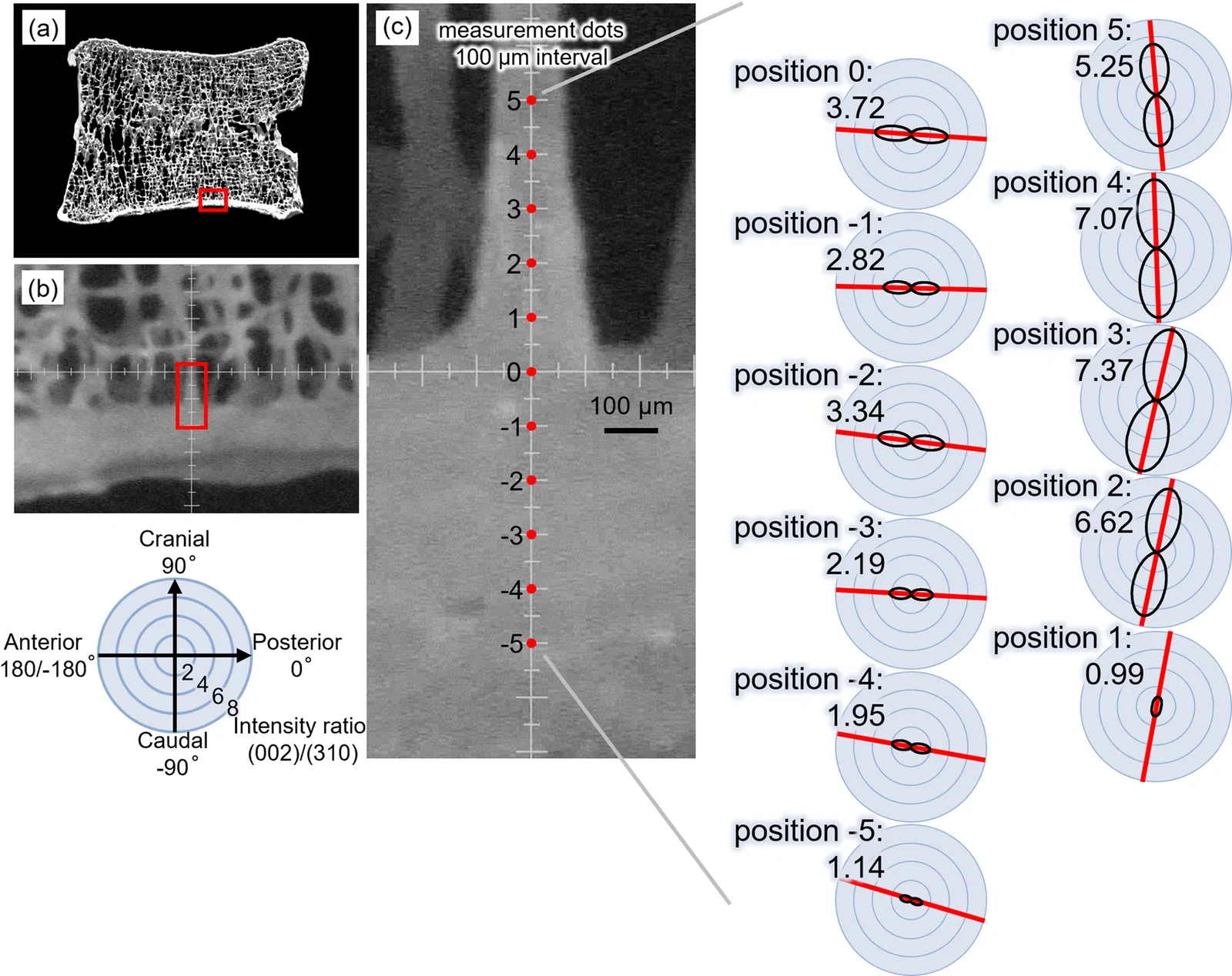
Local BAp *c*-axis orientation across the caudal corticotrabecular junction in Specimen 1. **a** Sagittal CT image. **b** Magnified view of the analyzed region. **c** Higher-magnification view with local orientation analysis at 100-µm intervals. The black ellipse represents the fitted angular distribution of the (002)/(310) intensity ratio, and the red line indicates the preferred BAp *c*-axis direction. The numerical value beside each plot indicates the maximum fitted (002)/(310) intensity ratio. A localized decrease in the maximum fitted (002)/(310) intensity ratio, accompanied by a change in the preferred BAp *c*-axis direction, was observed at the corticotrabecular junction

Based on the above findings, preferred BAp *c*-axis orientation in human vertebrae generally followed the local cortical surface orientation in the sagittal plane. Because the subchondral bone on the cranial and caudal surfaces intersects the underlying trabeculae nearly perpendicularly, we hypothesized that an orientation mismatch could arise at such interfaces. We therefore examined whether the preferred orientation remains continuous across the corticotrabecular junction or changed abruptly when traversing it. Figure 3 shows the maximum BAp (*c*-axis) orientation values and directions at the junction in Specimen 1. At the caudal corticotrabecular junction, the endplate-adjacent cortical region exhibited anteroposterior orientation, whereas the adjacent trabecular bone showed craniocaudal orientation. A marked local change in the preferred BAp *c*-axis direction was observed across the corticotrabecular junction. This change occurred between adjacent measurement positions separated by 100 μm, indicating that the transition occurred within a spatial range of 100 μm or less. The maximum fitted intensity ratio of (002)/(310) value at the corticotrabecular junction was lower than that in the adjacent trabecular bone, indicating weaker preferential BAp *c*-axis alignment at the junction.

### Principal Stress Distribution of Vertebrae and Its Directional Correspondence with BAp Orientation

**Fig. 4:**
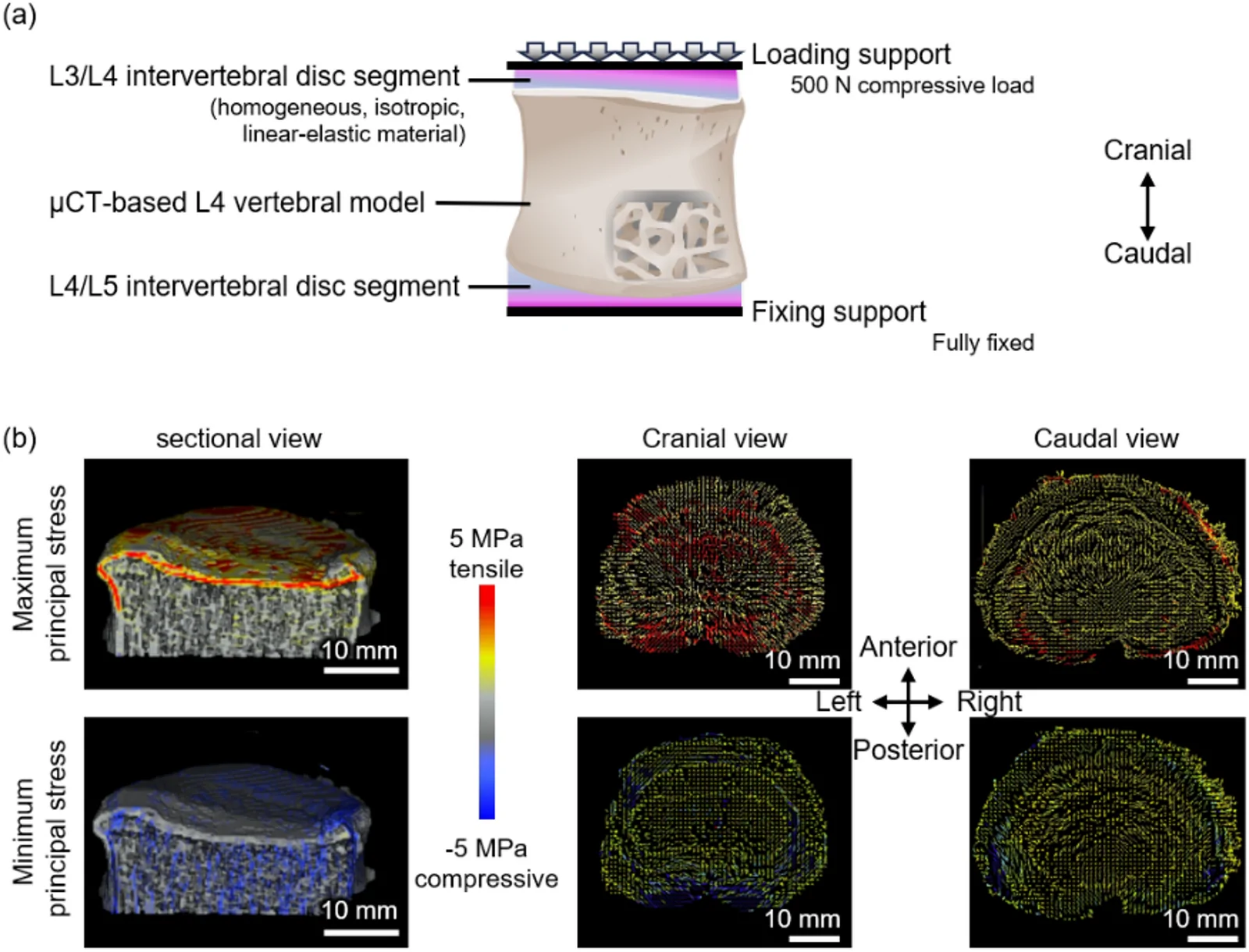
Finite element model and principal stress distribution in the human lumbar vertebra. **a** Schematic of the µCT-based FE model consisting of a single L4 vertebral body with the adjacent L3/L4 and L4/L5 intervertebral disc segments. The disc segments were modeled as homogeneous, isotropic, linear-elastic materials. A 500-N compressive load was applied through the cranial loading support, whereas the caudal fixing support was fully constrained. **b** Maximum and minimum principal stress distributions in Specimen 1. The upper and lower rows show the maximum and minimum principal stresses, respectively. Sagittal sectional, cranial, and caudal views of the same L4 vertebral model are shown. Positive and negative values indicate tensile and compressive principal stresses, respectively.

To characterize the local mechanical environment, finite element analysis (FEA) was performed based on µCT-derived geometry. The model employed simplified isotropic linear-elastic material properties to evaluate externally generated stress fields without introducing orientation-derived anisotropy into the material definition. Figure 4 shows the FE model and the maximum and minimum principal stress distributions in Specimen 1. Although axial compression was applied globally along the craniocaudal direction, the local principal stress state predicted by the FE model varied among anatomical regions. Compressive principal stresses were predominantly observed in the anterior region, whereas local tensile principal stresses were observed in the cranial and caudal regions.

**Fig. 5:**
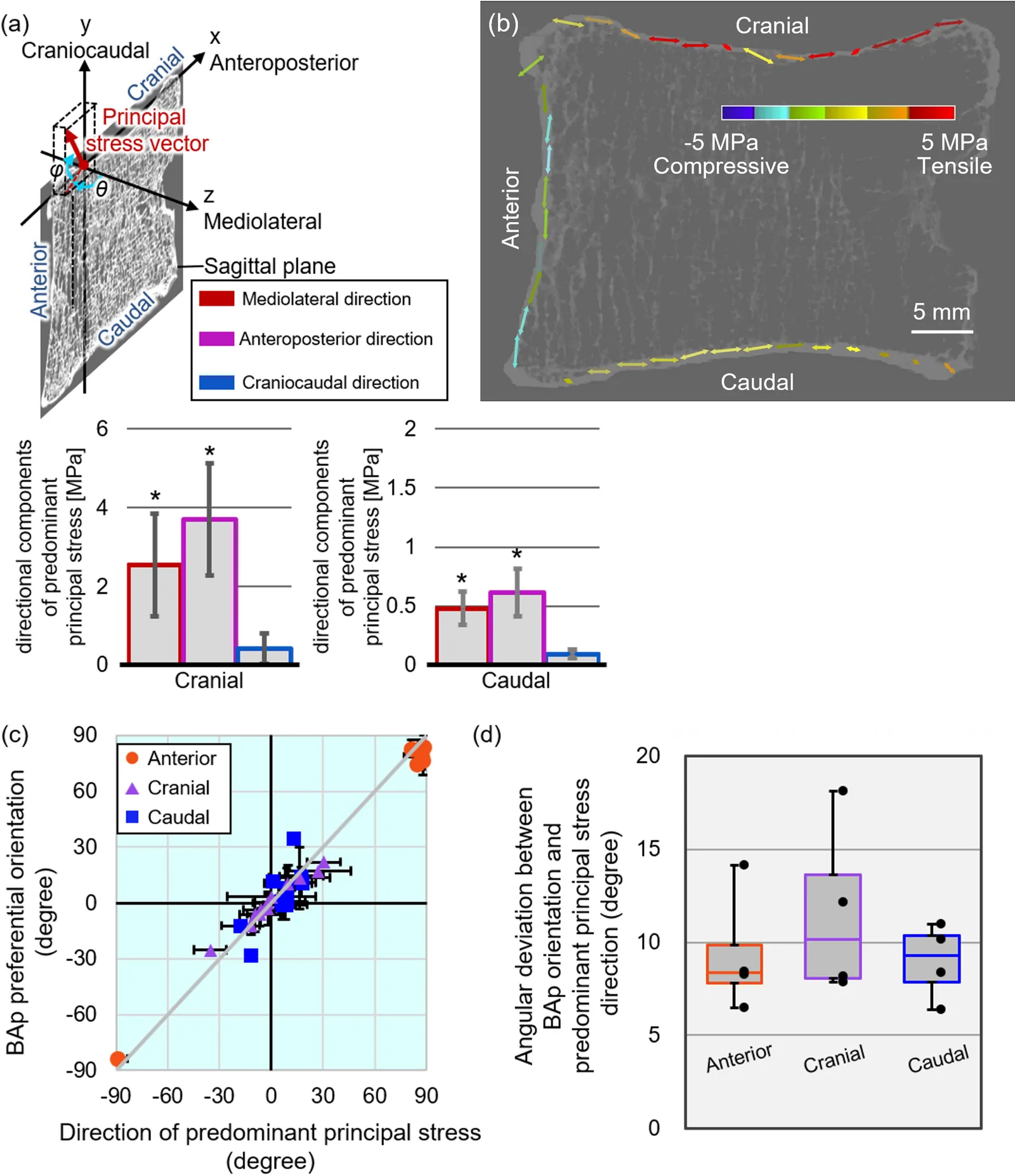
Directional correspondence between principal stress and BAp orientation. **a** Directional components of the predominant principal stress in the cranial and caudal regions. At each XRD-corresponding FE location, the predominant principal stress was resolved into the mediolateral, anteroposterior, and craniocaudal components. Data are presented as mean ± SD across the four vertebrae. Statistical differences were evaluated by two-way repeated-measures ANOVA with anatomical region and direction as within-specimen factors, followed by pairwise comparisons among directions within each region with adjustment for multiple comparisons. *: *P* < 0.05 vs. craniocaudal direction within the same region. **b** Predominant principal stress directions projected onto the sagittal plane in Specimen 1. The three-dimensional predominant principal stress direction was represented as a unit vector before projection; therefore, the length of each projected arrow represents the magnitude of its sagittal-plane component, with longer arrows indicating directions closer to the sagittal plane and shorter arrows indicating directions closer to the plane normal. Arrow color indicates the value and sign of the predominant principal stress (positive, tensile; negative, compressive). **c** Relationship between the preferred BAp *c*-axis orientation measured by transmission-type XRD and the projected predominant principal stress direction. Each point represents the specimen-averaged value at each corresponding measurement position, and the gray line indicates y = x. **d** Angular deviation (Δ*θ*) between the preferred BAp *c*-axis orientation and the projected predominant principal stress direction in the anterior, cranial, and caudal regions. Each point represents the specimen-level mean Δ*θ* within the corresponding anatomical region. Smaller values indicate closer directional correspondence.

Because the cranial and caudal regions showed a two-dimensional BAp orientation pattern within the anteroposterior–mediolateral plane, we next examined the three-directional components of the predominant principal stress in these regions (Fig. 5a). The predominant principal stress was resolved into the anteroposterior, craniocaudal, and mediolateral components and evaluated in the cranial and caudal regions. Two-way repeated-measures ANOVA revealed a significant interaction between anatomical region and direction (*P* = 0.015). Within the cranial region, the anteroposterior and mediolateral components were significantly greater than the craniocaudal component (adjusted *P* = 0.0482 for both comparisons). Similarly, within the caudal region, both the mediolateral and anteroposterior components were significantly greater than the craniocaudal component (adjusted *P* = 0.0197 for both comparisons). These results indicate that, in both regions, the predominant principal stress was oriented preferentially within the anteroposterior–mediolateral plane rather than along the craniocaudal axis.

We then examined the local directional correspondence between the predominant principal stress direction and the preferred BAp orientation obtained by transmission-type XRD within the sagittal plane. For this separate analysis, the three-dimensional predominant principal stress direction at each XRD-corresponding FE location was orthogonally projected onto the sagittal plane (Fig. 5b). The projected predominant principal stress directions showed region-specific distributions: the anterior region was predominantly aligned along the craniocaudal axis, whereas the cranial and caudal regions were predominantly oriented along the anteroposterior direction within the sagittal plane.

The projected predominant principal stress direction was subsequently compared directly with the preferred BAp orientation at the corresponding measurement locations. In Fig. 5c, the specimen-averaged values at each measurement position were distributed close to the identity line, indicating descriptive positional correspondence between preferred BAp orientation and predominant principal stress direction. Consistently, the angular deviation (Δ*θ*) between the two directions remained relatively small across the anatomical regions (Fig. 5d), further supporting local directional correspondence between BAp orientation and the predominant principal stress direction predicted by FEA. This directional correspondence was also consistent with the local cortical architecture. Taken together, these results demonstrate region-specific correspondence among predominant principal stress direction, cortical architecture, and BAp orientation at spatially matched vertebral locations.

Finally, specimen-level distributions of local principal stress magnitude and BAp orientation in the anterior region are summarized in Fig. S3. A lower-BMD specimen exhibiting markedly elevated local principal stress also showed relatively high BAp orientation at several measurement positions; however, because of the limited number of specimens, this observation was considered descriptive.

## Discussion

**Fig. 6:**
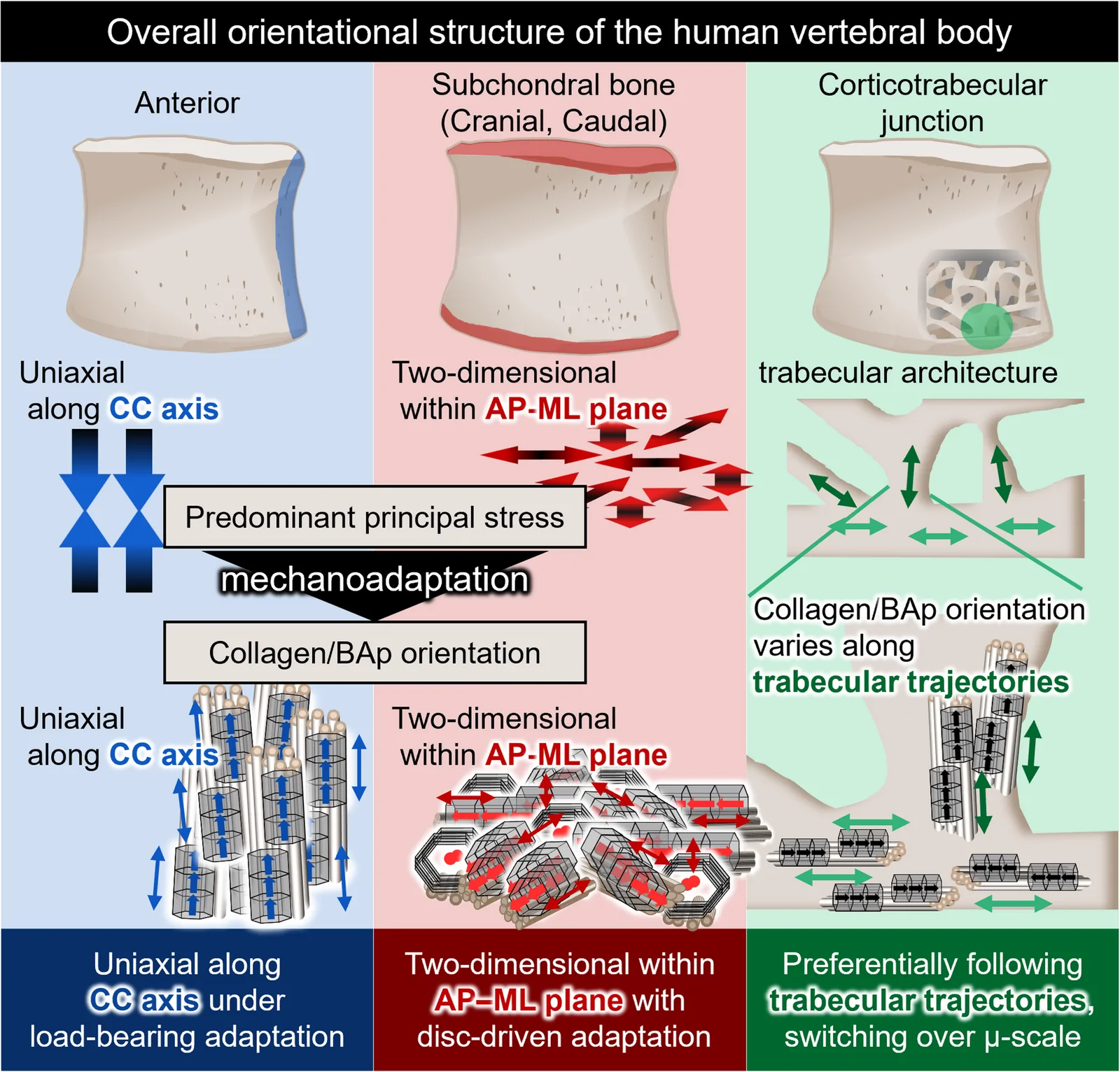
Schematic summary of region-specific correspondence between BAp orientation and predominant principal stress direction in the human lumbar vertebral body. The anterior cortex shows predominantly uniaxial craniocaudal BAp alignment corresponding to craniocaudally directed stress, whereas the cranial and caudal subchondral regions show a biaxial in-plane orientation pattern within the anteroposterior–mediolateral plane corresponding to principal stress directions within that plane. An abrupt transition in preferred orientation is observed at the corticotrabecular junction

To our knowledge, this study is the first to show a region-specific directional correspondence between BAp crystal orientation and the local predominant principal stress direction estimated by FEA in human lumbar vertebrae (Fig. 6). The anterior cortical region exhibited predominantly uniaxial alignment along the craniocaudal axis, corresponding to a predominantly craniocaudally directed principal stress. In contrast, the cranial and caudal subchondral regions showed a two-dimensional in-plane orientation pattern within the anteroposterior–mediolateral plane, corresponding to principal stress directions preferentially distributed within the same plane. At the examined corticotrabecular junction in Specimen 1, the preferred BAp orientation changed markedly between adjacent measurement positions separated by 100 μm.

The macroscopic structure of cancellous bone is believed to develop along principal stress directions, as described by Wolff’s law [24]. Similarly, the preferential alignment direction of fine crystals has been reported to follow the long axes of bones and trabeculae [25]. In the current study, cortical bone analysis revealed uniaxial *c*-axis alignment along the craniocaudal axis in the anterior region, whereas a two-dimensional preferential orientation was observed on the cranial and caudal surfaces within the anteroposterior–mediolateral plane. Although collagen fiber orientation was not directly quantified in the present specimens, collagen and BAp are generally co-aligned in bone such that the apatite *c*-axis tends to run parallel to the collagen fibril axis [8, 26].

The trabecular XRD measurements in the present study were designed to characterize the local change in BAp orientation across the corticotrabecular junction rather than to establish a quantitative site-matched relationship with the local FE stress state. Although the measurement positions were spatially defined, the diffraction sampling volume in trabecular bone does not necessarily correspond directly to the three-dimensional local volume used for FE stress evaluation. Therefore, the trabecular BAp measurements were not directly paired with local principal stress values in the present analysis. The observed BAp orientation accords with prior work showing that cancellous-bone anisotropy generally aligns with trabecular trajectories at the local level [27, 28]. We further demonstrate that crystallographic orientation can change over a highly localized spatial interval at structural interfaces, as indicated by the localized transition at the subchondral cortex–primary trabecula junction. Together, these findings suggest that similarly steep orientation changes may also occur at other vertebral interfaces where trabecular trajectories and load-transfer pathways change, such as at the anterior cortical shell–secondary trabecula junction, although those regions were not directly measured in the present study.

These findings can also be interpreted within a hierarchical mechanobiological framework linking local mechanical states to anisotropic matrix organization. Osteocytes are regarded as primary mechanosensors in bone. An osteocyte–osteoblast mechano-coculture system has demonstrated that osteocyte responses to oscillatory mechanical stimuli regulate osteoblast arrangement via soluble factors, thereby influencing subsequent oriented collagen/apatite organization [29]. Consistent with this concept, the degree of osteoblast alignment has been shown to quantitatively govern the subsequent orientation of the collagen/apatite matrix produced by the cells [30]. Moreover, osteocyte lacuno-canalicular anisotropy has been reported to correlate with apatite orientation anisotropy, supporting a coupling between osteocyte organization and anisotropic extracellular matrix architecture [31]. Together with evidence that apatite orientation can adaptively increase under elevated in vivo loading [25], the present stress–orientation correspondence in human vertebrae is consistent with this hierarchical model, although the pathway itself was not directly tested in this study. Recent evidence further suggests that anisotropic bone quality may also be influenced by specific molecular determinants. For example, osteocalcin deficiency has been reported to disrupt BAp *c*-axis orientation and reduce bone strength without altering collagen orientation, supporting the concept that factors regulating crystallographic anisotropy may serve as important determinants of bone quality [32].

Previous studies have evaluated vertebral stress distributions under intervertebral disc- or disc-like tissue-mediated loading using µCT-based finite element analysis [33, 34], and Rokita et al. further demonstrated a relationship between hydroxyapatite crystallite orientation and FE-derived stress distribution in human vertebral trabecular bone using micro-XRD [35]. The present study extends these findings by spatially matching BAp orientation with predominant principal stress directions derived from FE models reconstructed from the same vertebral specimens. The three-directional analysis showed that the predominant principal stress in the cranial and caudal regions had substantial components within the anteroposterior–mediolateral plane, corresponding to the two-dimensional BAp orientation pattern, while sagittal-plane projection further demonstrated local directional correspondence between the predominant principal stress and preferred BAp directions. This correspondence may reflect a mechanically rational adaptation in which alignment of the stiff BAp *c*-axis with the predominant principal stress direction reduces strain under a given stress.

Although axial compression was applied globally, local tensile principal stresses were observed in the cranial and caudal regions. Fields et al. similarly reported tensile strains near the vertebral endplates under disc-mediated compression [16], suggesting that transverse expansion of the intervertebral disc may contribute to this local stress state, although effects of vertebral geometry and boundary conditions cannot be excluded. From a translational perspective, mechanical performance and biocompatibility remain fundamental considerations in implant design [36, 37], but the present findings suggest that orientation-dependent bone quality may also warrant consideration. The observed correspondence between local predominant principal stress direction and BAp orientation provides a mechanistic perspective for considering how implant-mediated mechanical environments may influence collagen/apatite orientation and, ultimately, functional bone quality around implants [38, 39].

A vertebra with lower BMD and markedly elevated local principal stress also exhibited relatively high BAp orientation at several measurement positions. This observation is consistent with the possibility, suggested by previous animal studies, that matrix anisotropy may contribute to maintaining mechanical competence under conditions of reduced bone mass [9, 10]. However, because the present study included only four specimens and did not use a clinically defined osteoporosis grouping, this observation should be regarded as hypothesis-generating and cannot establish a relationship between osteoporosis and crystallographic orientation in human vertebrae.

This study has several limitations. First, only four human specimens were analyzed, which limits the statistical power and generalizability of the present findings. The study was not designed or sufficiently powered to evaluate osteoporosis-related differences, and the specimen-level observations regarding BMD, local stress, and BAp orientation should therefore be regarded as hypothesis-generating. Verification of whether reduced bone mass is accompanied by adaptive enhancement of matrix orientation will require a substantially larger cohort with appropriate clinical classification and consideration of potential confounding factors such as age and sex. Nevertheless, the region-specific directional correspondence between predominant principal stress direction and BAp orientation was observed across the four specimens and should be interpreted as quantitative but descriptive, requiring validation in a larger cohort. Second, the FE-derived local predominant principal stress directions may depend on assumptions used in the model definition. Although variations in material properties under predominantly uniaxial compression are expected to affect stress magnitude more strongly than principal stress direction, a formal sensitivity analysis was not performed in the present study. Therefore, the robustness of the predicted stress directions remains to be established. Nevertheless, the regional stress patterns were consistent with previous biomechanical findings in human vertebrae under disc-mediated compressive loading, supporting the mechanical plausibility of the present FE results. Third, although BAp orientation was evaluated along three anatomical directions using transmission- and reflection-type XRD, the measurement positions were confined to a single midsagittal section of each vertebra. Therefore, the present analysis provides three-directional orientation information but does not constitute a fully three-dimensional spatial mapping of BAp orientation throughout the vertebral body. Future studies incorporating three-dimensional spatial sampling would enable a more comprehensive assessment of vertebral crystallographic organization. Finally, collagen fiber orientation or osteocyte morphology/organization were not quantified at corresponding microanatomical locations, because such site-matched evaluation would require a larger number of human vertebral specimens, which are difficult to obtain. Direct integration of these matrix- and cell-level parameters would enable more explicit testing of the proposed mechanobiological pathway in human vertebrae. Thus, the proposed mechanobiological pathway remains interpretive rather than directly demonstrated in the present specimens. Furthermore, factors other than orientation—such as apatite crystallite size/shape, mineral amount, and water content—have also been reported to contribute to bone mechanical competence [40, 41]. Although the present work focuses on crystallographic orientation because of its strong association with mechanical competence [8], integrating these complementary factors will provide a more comprehensive understanding in future work.

## Conclusions

This study identified a region-specific directional correspondence between BAp *c*-axis orientation and local predominant principal stress direction in human vertebrae. The anterior cortex showed predominantly uniaxial craniocaudal alignment corresponding to a predominantly craniocaudally directed principal stress, whereas the cranial and caudal subchondral regions showed a two-dimensional in-plane orientation pattern corresponding to principal stress directions within the anteroposterior–mediolateral plane. At the examined corticotrabecular junction, a marked change in BAp orientation was observed between adjacent measurement positions separated by 100 μm, indicating that the transition occurred within a spatial range of 100 μm or less. Taken together, these findings suggest a bone-quality–based perspective on vertebral mechanical competence and may inform future bone-quality–oriented structural reconstruction, device design, and in vivo assessment of anisotropic bone quality, including insights into the molecular regulation of crystallographic alignment.

## Supplementary Information

Below is the link to the electronic supplementary material.

Supplementary Material 1
