# Supplementary material for: Apatite c-axis Orientation Tracks Disc-Mediated Local Principal Stress Directions in the Human Lumbar Vertebra
Source: Calcif Tissue Int. 2026 Oct 23;117(1):167. doi: 10.1007/s00223-026-01629-0 (PMC13694369; doi:10.1007/s00223-026-01629-0)
Supplement: Supplementary file 1 [file 223_2026_1629_MOESM1_ESM.docx]

**Apatite *c*-axis orientation tracks disc-mediated local principal stress directions in the human lumbar vertebra**

Toko Mori *^a^*^#^, Tadaaki Matsuzaka *^a^*^#^, Aira Matsugaki *^a^*, Sayaka Miyabe *^a^*, Nobuhito Nango *^b^*, Yukihide Minoda *^c^*, Hiroyoshi Iwaki *^c,d^*, Akio Kobayashi *^c,e^*, Takayoshi Nakano *^a*^*

*^a^ Division of Materials and Manufacturing Science, Graduate School of Engineering, The University of Osaka, Japan*

*^b^ R&D Department, Ratoc System Engineering Co., Ltd, Japan*

*^c^ Department of Orthopaedic Surgery, Osaka Metropolitan University, Graduate School of Medicine, Japan*

*^d^ Department of Orthopaedic Surgery, Osaka Orthopedic Hospital, Osaka, Japan*

*^e^ Department of Orthopaedic Surgery, Shiraniwa Hospital, Nara, Japan*

^#^: These authors contributed equally to this work.

***Corresponding author**

Takayoshi Nakano

[nakano@mat.eng.osaka-u.ac.jp](mailto:nakano@mat.eng.osaka-u.ac.jp)

*Division of Materials and Manufacturing Science, Graduate School of Engineering, The University of Osaka, 2-1, Yamada-Oka, Suita, Osaka 565-0871, Japan*

Tel./Fax: +81-(0)6-6879-7505

**Table S1 |** Material properties applied for finite element modeling

| Region |  | Representative density  (mg/cm^3^) | Young’s modulus  (MPa) | Poisson’s ratio  (-) |
| --- | --- | --- | --- | --- |
| Vertebral body | Cortical bone | 1100 | 22000 [13] | 0.30 [20] |
|  | Trabecular bone | 600 | 3500 [14] | 0.30 [20] |
|  | Low-density | 300 | 440 [15] | 0.30 [20] |
| Intervertebral disc |  | ― | 8 [18] | 0.45 [19] |

Representative density were used solely as reference density labels for discretized material classes and were not treated as continuous density-dependent material parameters.
The low-density region includes marrow space and partial-volume voxels below the trabecular threshold; it does not represent soft biological tissue.

**Fig. S1** | Analysis points of BAp orientation, trabecular trajectory, and stress direction in the anterior, cranial, and caudal cortical regions of representative Specimen 1 (a) and Specimen 3 (b) vertebrae. Red dots indicate the XRD measurement positions.

**Fig. S2** | Interpretation of the polar fitting used to determine BAp *c*-axis orientation. (a) Schematic representation of the angular distribution of the (002)/(310) intensity ratio. Orange points indicate the measured intensity ratios at individual measurement angles ($\theta$), and the fitted curve represents the elliptical fit to the angular distribution. The radial coordinate $r\left( \theta\right)$represents the fitted (002)/(310) intensity ratio at angle $\theta$. The maximum fitted value ($r_{\max}$) was defined as the BAp orientation value, and its corresponding angular direction ($\alpha$) as the preferred BAp *c*-axis direction. (b) Representative distribution showing a higher BAp orientation value ($r_{\max}=1.4$) and clear angular dependence. (c) Representative distribution with $r_{\max}=0.6$, showing weak angular dependence. The fitted distribution represents the angular dependence of the diffraction intensity ratio and does not represent the physical shape of the apatite crystals.

**Fig. S3** | Specimen-level distributions of local principal stress magnitude and BAp orientation in the anterior region. Local principal stress magnitude and BAp orientation value at the corresponding measurement positions in the four individual specimens. Bars represent the values for each specimen at each measurement position, with the same specimen indicated consistently between panels.
